# Inhibition of Casein Kinase 2 Modulates XBP1-GRP78 Arm of Unfolded Protein Responses in Cultured Glial Cells

**DOI:** 10.1371/journal.pone.0040144

**Published:** 2012-06-29

**Authors:** Toru Hosoi, Kenta Korematsu, Naohiro Horie, Takahiro Suezawa, Yasunobu Okuma, Yasuyuki Nomura, Koichiro Ozawa

**Affiliations:** 1 Department of Pharmacotherapy, Graduate School of Biomedical and Health Sciences, Hiroshima University, Hiroshima, Hiroshima, Japan; 2 Department of Pharmacology, Graduate School of Pharmaceutical Sciences, Hokkaido University, Sapporo, Hokkaido, Japan; 3 Department of Pharmacology, Faculty of Pharmaceutical Sciences, Chiba Institute of Sciences, Choshi, Chiba, Japan; 4 Department of Pharmacotherapeutics, Yokohama College of Pharmacy, Yokohama, Kanagawa, Japan; University of Hong Kong, Hong Kong

## Abstract

Stress signals cause abnormal proteins to accumulate in the endoplasmic reticulum (ER). Such stress is known as ER stress, which has been suggested to be involved in neurodegenerative diseases, diabetes, obesity and cancer. ER stress activates the unfolded protein response (UPR) to reduce levels of abnormal proteins by inducing the production of chaperon proteins such as GRP78, and to attenuate translation through the phosphorylation of eIF2α. However, excessive stress leads to apoptosis by generating transcription factors such as CHOP. Casein kinase 2 (CK2) is a serine/threonine kinase involved in regulating neoplasia, cell survival and viral infections. In the present study, we investigated a possible linkage between CK2 and ER stress using mouse primary cultured glial cells. 4,5,6,7-tetrabromobenzotriazole (TBB), a CK2-specific inhibitor, attenuated ER stress-induced XBP-1 splicing and subsequent induction of GRP78 expression, but was ineffective against ER stress-induced eIF2α phosphorylation and CHOP expression. Similar results were obtained when endogenous CK2 expression was knocked-down by siRNA. Immunohistochemical analysis suggested that CK2 was present at the ER. These results indicate CK2 to be linked with UPR and to resist ER stress by activating the XBP-1-GRP78 arm of UPR.

## Introduction

Stress signals, which impair endoplasmic reticulum (ER) function, result in the accumulation of unfolded proteins. Such an accumulation causes ER stress. Increasing evidence has suggested that ER stress is involved in several types of disease including neurodegenerative disorders, diabetes, obesity and cancer. It is thus important to elucidate the precise mechanisms of ER stress-mediated activation of the unfolded protein response (UPR).

When exposed to ER stress, cells activate several UPR pathways. These responses include 1) increasing the folding capacity of unfolded proteins by releasing chaperon proteins, 2) inhibiting general protein translation to stop the production of unfolded proteins, and 3) promoting the degradation of unfolded proteins [1]–[3]. However, when exposed to severe stress, cells activate apoptotic pathways. As components responsible for the activation of UPR, several ER stress-sensing proteins, which reside in the ER, have been identified: i.e. inositol-requiring protein-1 (IRE1), PKR-like ER kinase (PERK), and activating transcription factor 6 (ATF6). Activation of these stress-sensors eventually transmits stress signals to the nucleus [4]. For example, activation of IRE1 induces X-box binding protein 1 (XBP-1) mRNA splicing [5]. The spliced form of XBP-1 then functions as a transcription factor for ER stress-related genes such as the glucose-regulated protein 78 (GRP78) gene [6]. GRP78 functions as a chaperon protein, involved in protein folding. The activation of PERK increases phosphorylation of the α subunit of eukaryotic translation initiation factor 2 (eIF2), resulting in translational repression [7], [8]. Meanwhile, the increase in eIF2 phosphorylation, paradoxically activates the CCAAT/enhancer-binding protein homologous protein (CHOP) promoter and results in production of CHOP, an apoptotic transcription factor [9].

Protein kinase CK2 is a serine/threonine protein kinase composed of two catalytic α, α’ subunits and two regulatory β subunits [10]. CK2 is involved in protecting cells from various kinds of stress. For example, UV irradiation increases CK2-dependent phosphorylation of p53, which would decrease the proapoptotic function of p53 [11]. Heat shock stress has been shown to re-localize CK2 subunits to specific nuclear regions [12]. Furthermore, stress-activating agents such as anisomycin, arsenite, and tumor necrosis factor-α (TNF-α) stimulate CK2 activity through p38 MAP kinase [13]. These observations suggest that CK2 plays an important role in protecting cells against such stress. However, it is unknown whether CK2 is involved in protecting against type of stress, which perturb ER function (ER stress). In the present study, therefore, we investigated the possible role of CK2 under ER stress.

## Results

### CK2 Regulates ER Stress-induced Activation of the XBP-1-GRP78 Arm of UPR

**Figure 1:**
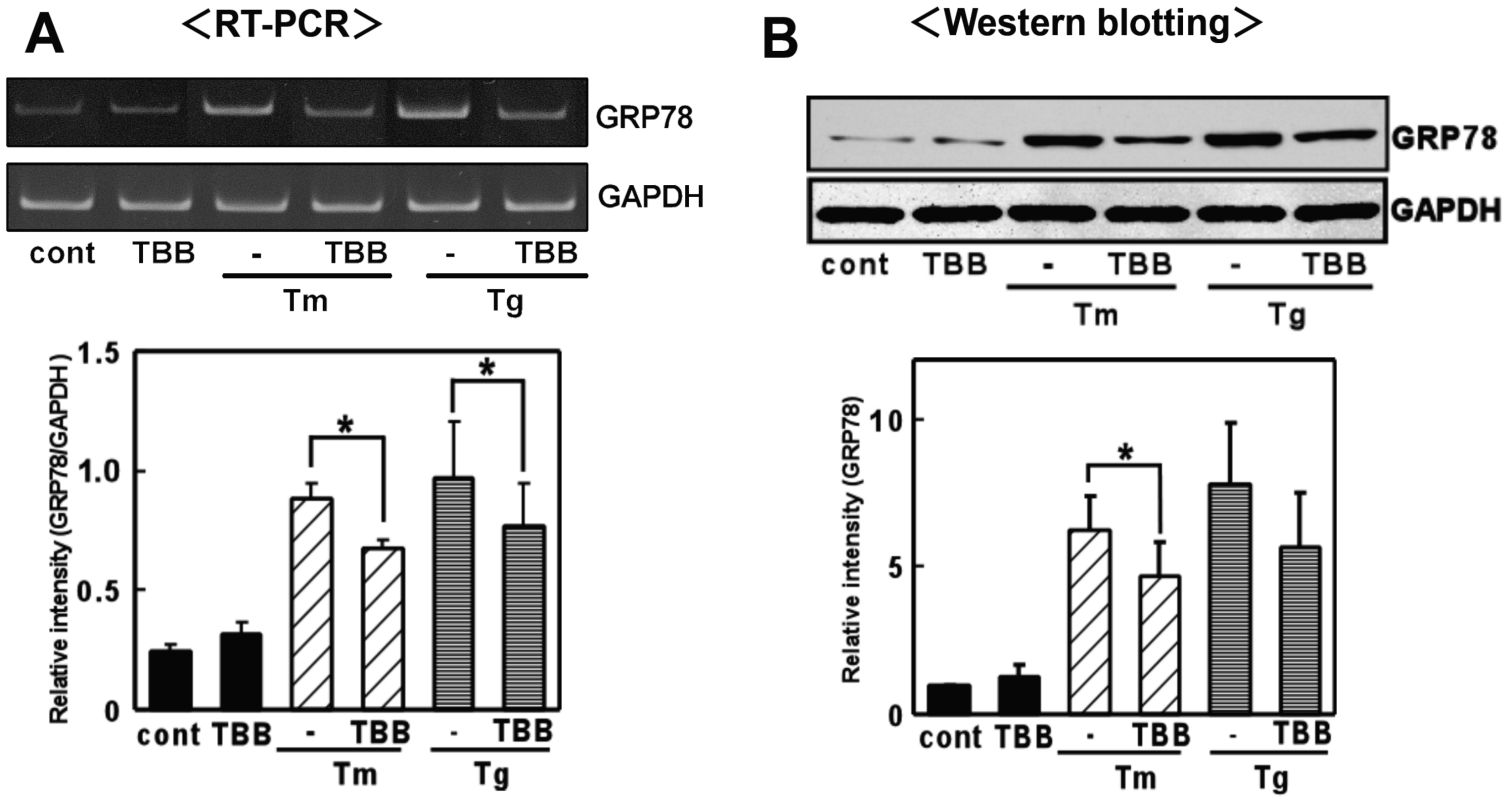
A CK2 inhibitor inhibited ER stress-induced GRP78 expression. 4,5,6,7-tetrabromobenzotriazole (TBB) inhibited GRP78 expression at the mRNA and protein levels. (A) Primary cultured glial cells were pre-treated with 4,5,6,7-tetrabromobenzotriazole (TBB: 5 µM) for 3 h and then treated with tunicamycin (Tm: 0.01 µg/mL) or thapsigargin (Tg: 0.01 µM) for 6 h. RT-PCR was performed using specific primers for each mRNA. n = 4/group *p<0.05 compared with ER stress (Tm, Tg) alone. (B) Primary cultured glial cells were pre-treated with 4,5,6,7-tetrabromobenzotriazole (TBB: 5 µM) for 3 h, then treated with tunicamycin (Tm: 0.01 µg/mL) or thapsigargin (Tg: 0.01 µM) for 18 h, and subjected to Western blotting. n = 3/group *p<0.05 compared with ER stress (Tm) alone. Results are expressed as the means ± S.E.

**Figure 2:**
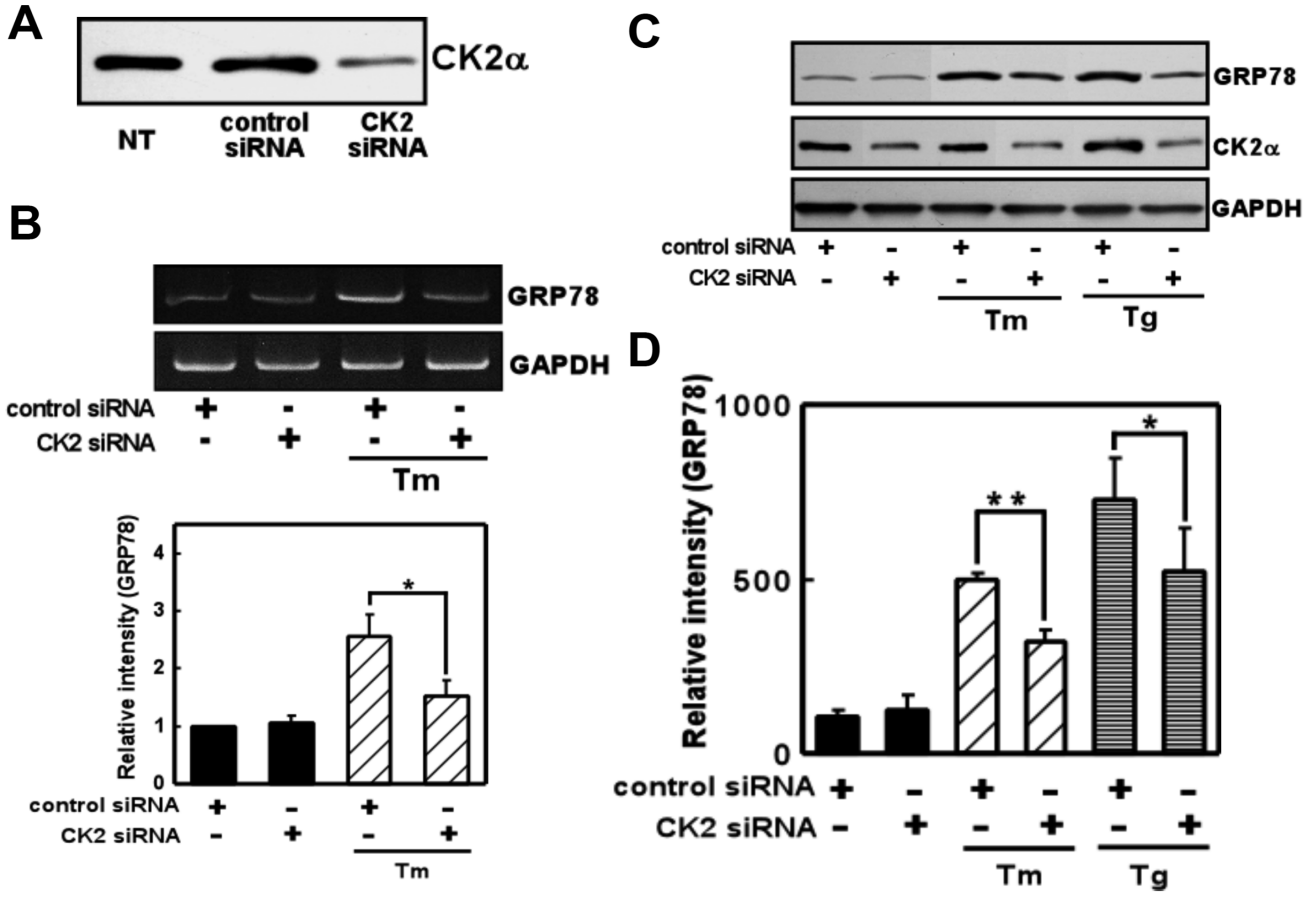
Knocking down CK2 inhibited ER stress-induced GRP78 expression. (A) Western blot analysis of endogenous CK2α expression in lysates of nontransfected cells (NT) and cells transfected with (short interfering RNA) siRNAs (75 nM) directed at CK2 or the control sequence. CK2 siRNA reduced the expression of CK2 compared with NT or control siRNA. (B) CK2 siRNA decreased ER stress-induced GRP78 expression compared with control siRNA. Primary cultured glial cells were transfected with 75 nM siRNA and treated with tunicamycin (Tm: 0.01 µg/mL) for 4 h. RT-PCR was then performed. n = 3/group (C, D) CK2 siRNA decreased ER stress-induced GRP78 expression compared with control siRNA. Primary cultured glial cells were transfected with 75 nM siRNA, treated with tunicamycin (Tm: 0.01 µg/mL) or thapsigargin (Tg: 0.01 µM) for 18 h, and subjected to Western blotting. n = 3–4/group *p<0.05, **p<0.01 compared with control siRNA. Results are expressed as the means ± S.E.

UPR was induced upon treatment with ER stress-inducing reagent in the glial cells [14]–[16]. Glial cells especially astrocyte have unique property to tolerate against ischemic or hypoxic stress, which lead to ER stress. One of the responsive mechanisms of the resistance against glial cell death would be mediated through the old astrocyte specifically induced substance (OASIS) [17]. In the present study, we did not observe prominent glial cell death as far as we can ascertain in the present condition. To evaluate the role of CK2 in the ER stress-induced activation of UPR, we exposed glial cells to ER stress-inducing reagents (tunicamycin: Tm, which interferes with protein glycosylation, and thapsigargin: Tg, which interferes with the Ca^2+^ balance) along with the CK2-specific inhibitor 4,5,6,7-tetrabromobenzotriazole (TBB) [18], and examined the level of GRP78. Consistent with a previous report [14], the expression of GRP78 was induced by the reagents in primary cultured glial cells (Fig. 1). TBB treatment alone did not affect GRP78 levels (Fig. 1). However, the expression of GRP78 was inhibited by pre-treatment with TBB (Fig. 1). The inhibitory effects of TBB were observed at both the mRNA and protein levels (Fig. 1AB). To further confirm the contribution of CK2 to ER stress-induced GRP78 expression, we next knocked down endogenous CK2 expression by transfecting CK2 siRNA. The CK2 siRNA but not control siRNA markedly reduced the endogenous level of CK2 (Fig. 2A). Therefore, we next analyzed the expression of GRP78 under these conditions. Again, knocking down CK2 alone did not affect GRP78 levels (Fig. 2B-D). However, we observed significant inhibition of ER stress-induced expression of GRP78 at both the mRNA and protein levels on knocking down CK2 (Fig. 2B-D). Therefore, the results suggest CK2 to be involved in the ER stress-induced expression of GRP78.

**Figure 3:**
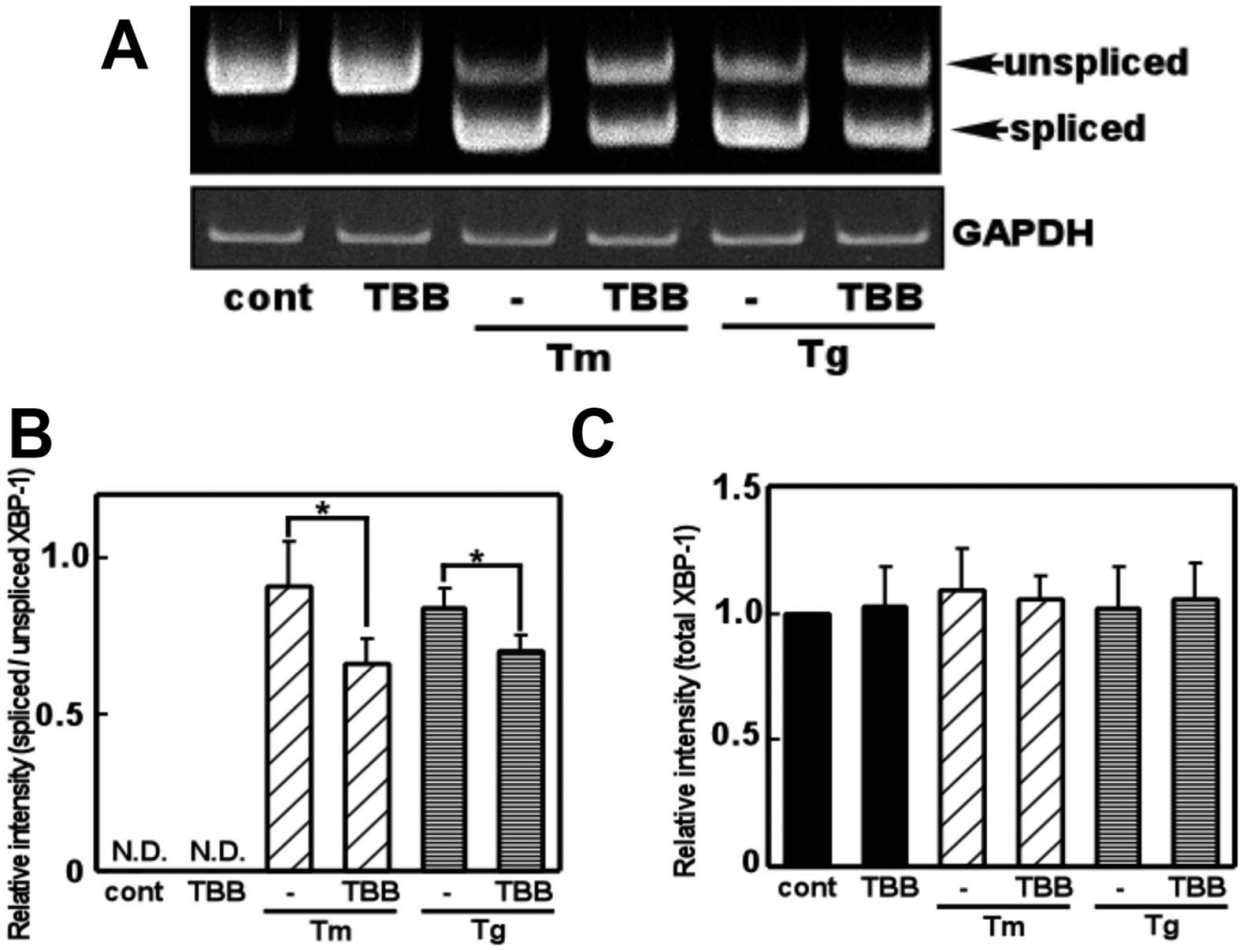
The CK2 inhibitor inhibited ER stress-induced XBP-1 mRNA splicing. (A) Primary cultured glial cells were pre-treated with 4,5,6,7-tetrabromobenzotriazole (TBB: 5 µM) for 3 h, treated with tunicamycin (Tm: 0.01 µg/mL) or thapsigargin (Tg: 0.01 µM) for 6 h, and subjected to a RT-PCR analysis. (B) Densitometric analysis of spliced/unspliced XBP-1 mRNA. An ER stress inducer increased XBP-1 splicing and this effect was significantly inhibited by TBB. n = 4–5/group *p<0.05 compared with ER stress (Tm, Tg) alone (C) Densitometric analysis of total XBP-1 mRNA (unspliced and spliced XBP-1 mRNA). n = 5/group. TBB did not affect the XBP-1 transcript. Results are expressed as the means ± S.E.

**Figure 4:**
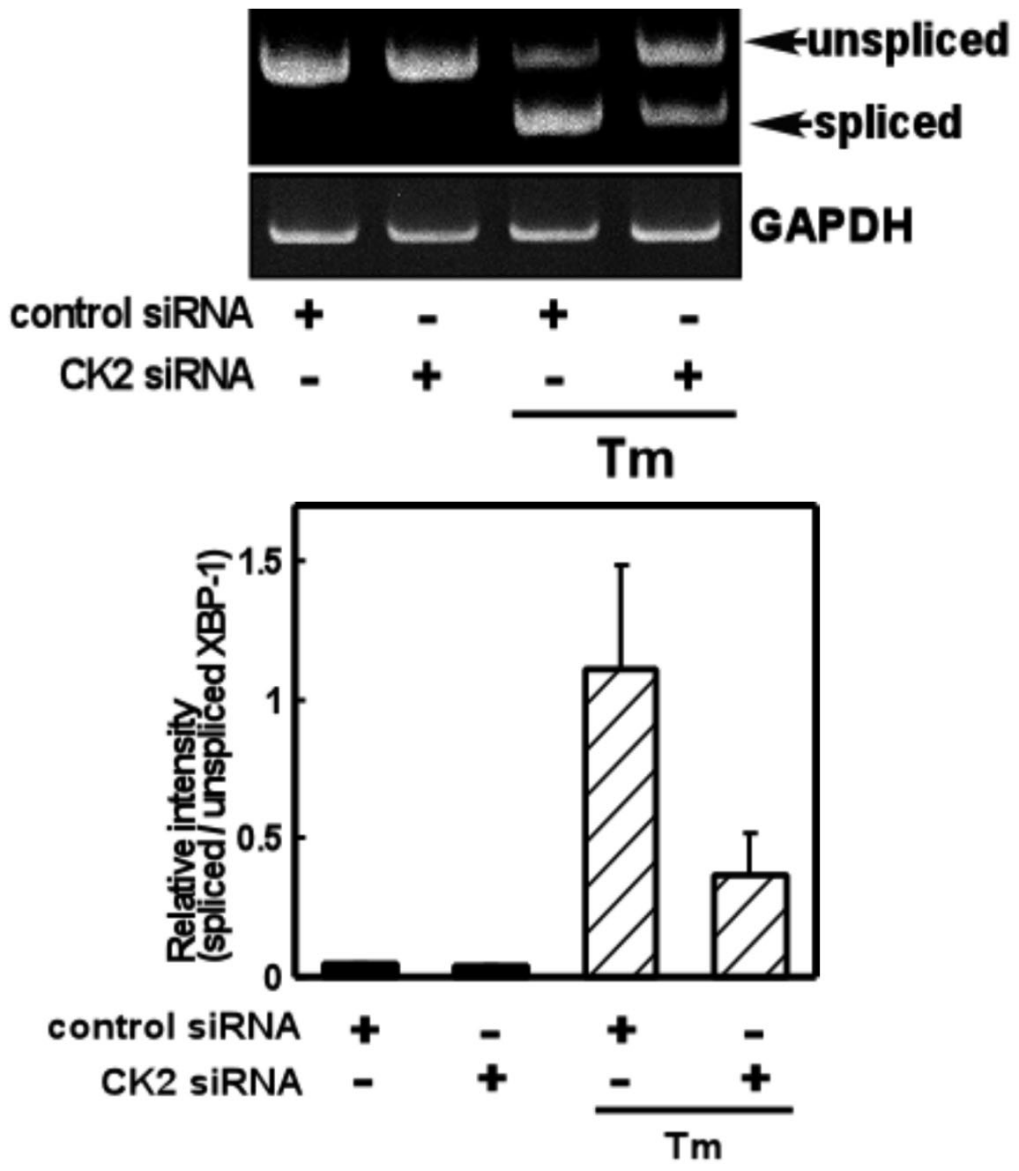
Knocking down CK2 inhibited ER stress-induced XBP-1 splicing. CK2 siRNA decreased ER stress-induced XBP-1 splicing compared with control siRNA. Primary cultured glial cells were transfected with 75 nM siRNA and treated with tunicamycin (Tm: 0.01 µg/mL) for 4 h. A RT-PCR analysis was then performed. n = 3/group. Results are expressed as the means ± S.E.

As we found that CK2 regulates GRP78 levels via the mRNA, we next focused on the upstream regulators of GRP78. Accordingly we investigated the role of CK2 in ER stress-induced splicing of XBP-1, an upstream regulator of GRP78 [6]. We treated glial cells with a CK2 inhibitor (TBB) and analyzed the level of XBP-1 splicing. We did not observe any changes in XBP-1 splicing on treatment with TBB alone, but observed significant inhibition of ER stress-induced XBP-1 splicing by TBB (Fig. 3AB). Conversely, the total amount of XBP-1 (spliced and unspliced XBP-1) was not changed by TBB, suggesting that CK2 did not affect the XBP-1 transcript (Fig. 3C). To further confirm these results, we next knocked down endogenous CK2 expression by transfecting CK2 siRNA and analyzed ER stress-induced XBP-1 splicing. Consistent with the results obtained using TBB, we observed inhibition of ER stress-induced XBP-1 splicing on knocking down CK2 (Fig. 4). Overall, from these observations, it is suggested that CK2 plays a key role in the ER stress-induced activation of the XBP-1-GRP78 arm of UPR.

### CK2 may not be Involved in Regulation of ER Stress-induced Activation of the eIF2α-CHOP Arm of UPR

**Figure 5:**
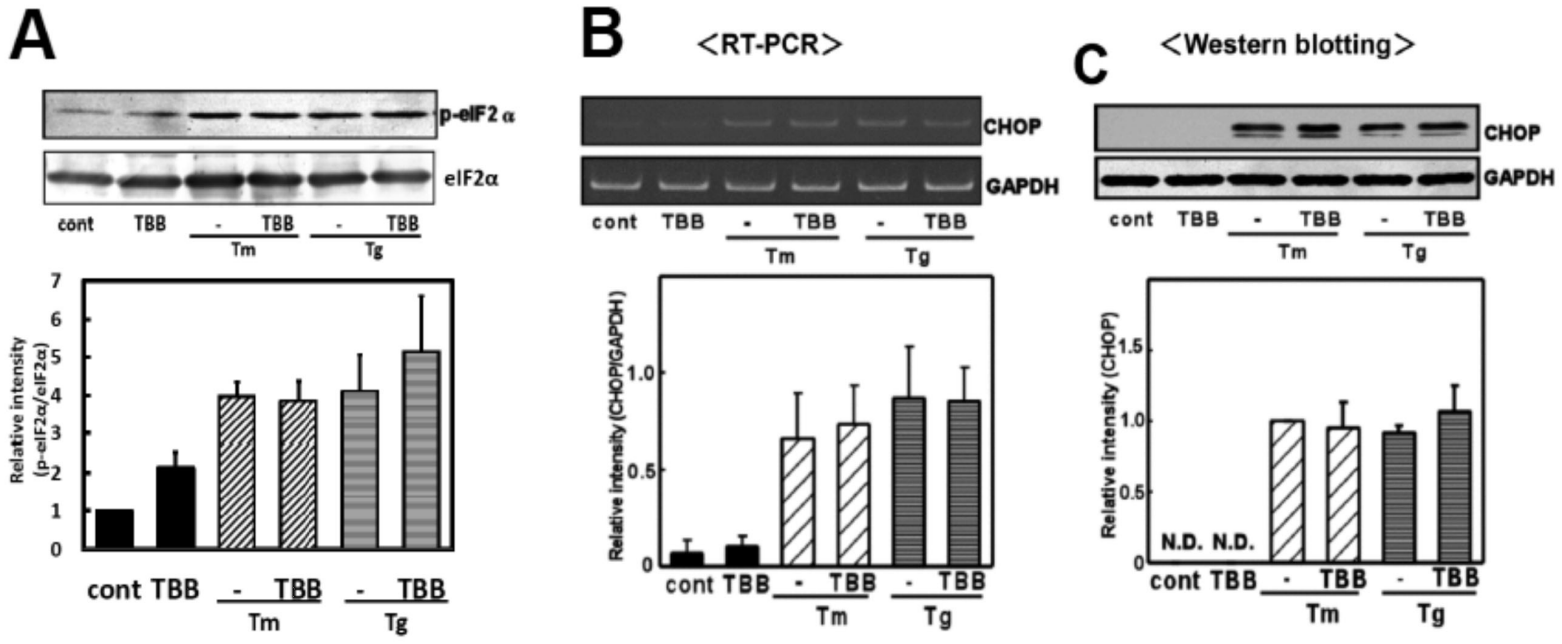
The CK2 inhibitor did not affect the eIF2α-CHOP arm of the ER stress-induced UPR. (A) Primary cultured glial cells were pre-treated with 4,5,6,7-tetrabromobenzotriazole (TBB: 5 µM) for 3 h and treated with tunicamycin (Tm: 0.01 µg/mL) or thapsigargin (Tg: 0.01 µM) for 4 h. Western blotting was then performed. TBB did not affect ER stress-induced eIF2α phosphorylation. Densitometric analysis of the normalization data of the phospho-eIF2α and eIF2α intensities were done. n = 3 per group. (B) Primary cultured glial cells were pre-treated with 4,5,6,7-tetrabromobenzotriazole (TBB: 5 µM) for 3 h and treated with tunicamycin (Tm: 0.01 µg/mL) or thapsigargin (Tg: 0.01 µM) for 6 h. A RT-PCR analysis was then performed. n = 3/group TBB did not affect ER stress-induced CHOP mRNA expression. (C) Primary cultured glial cells were pre-treated with 4,5,6,7-tetrabromobenzotriazole (TBB: 5 µM) for 3 h and treated with tunicamycin (Tm: 0.01 µg/mL) or thapsigargin (Tg: 0.01 µM) for 18 h. Western blotting was then performed. n = 4/group TBB did not affect ER stress-induced CHOP protein production. Results are expressed as the means ± S.E.

We next investigated the possible involvement of CK2 in another important arm of UPR, the eIF2α-CHOP pathway. We treated glial cells with TBB and analyzed the ER stress-induced phosphorylation of eIF2α, a downstream regulator of ER stress sensor protein PERK. As indicated in figure 5A, we observed no changes in the phosphorylation of eIF2α. To further confirm the results, we next investigated the effect of TBB on ER stress-induced expression of CHOP, a transcription factor known to be regulated through eIF2α [9]. We used TBB along with an ER stress-inducing reagent and analyzed the level of CHOP. As expected, we did not find any changes in CHOP at the mRNA or protein level after the treatment with TBB (Fig. 5BC). These findings indicate that CK2 would not affect ER stress-induced activation of the eIF2α-CHOP arm of UPR.

**Figure 6:**
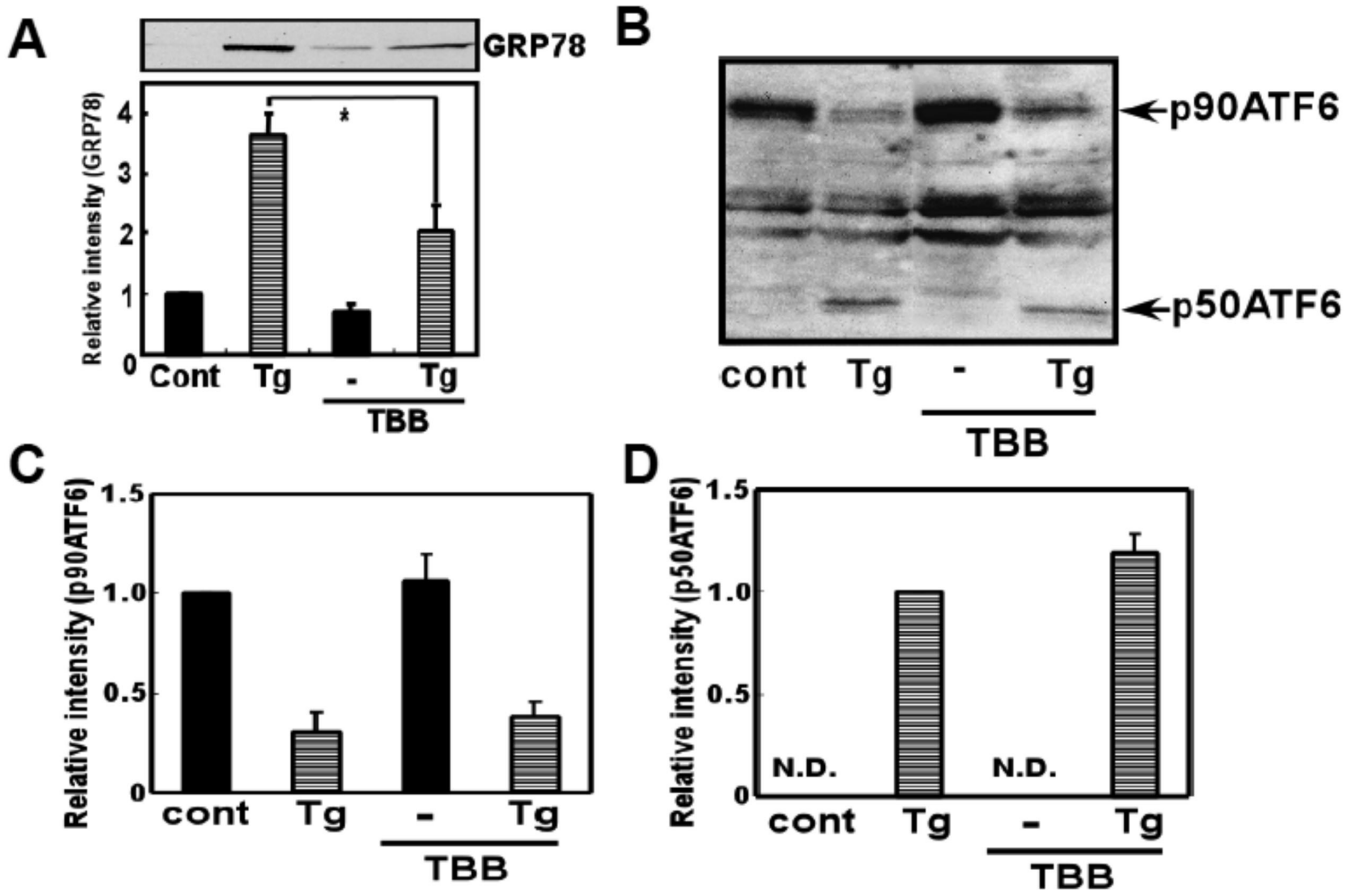
CK2 inhibitor did not affect ER stress-induced ATF6 processing. (A) SH-SY5Y cells were pre-treated with 4,5,6,7-tetrabromobenzotriazole (TBB: 5 µM) for 3 h, and then treated with thapsigargin (Tg: 10 µM) for 18 h and Western blotting analysis was performed. TBB inhibited ER stress-induced GRP78 expression. *p<0.05 (B) SH-SY5Y cells were pre-treated with 4,5,6,7-tetrabromobenzotriazole (TBB: 5 µM) for 3 h, and then treated with thapsigargin (Tg: 10 µM) for 4 h and Western blotting analysis was performed using ATF6 antibody. (C) Densitometric analysis of p90ATF6. (D) Densitometric analysis of p50 ATF6. TBB did not affect ER stress-induced ATF6 cleavage. n = 2–3/group. Results are expressed as the means ± S.E.

To further analyze possible involvement of CK2 on ATF6 pathway, the effect of TBB on ER stress-induced ATF6 cleavage was analyzed in SH-SY5Y cell line. Similarly with the result obtained from glial cells, we observed significant inhibition of ER stress-induced induction of GRP78 when treated with TBB (Fig. 6A). However, we did not observe inhibition of ER stress-induced ATF6 cleavage by TBB (Fig. 6B-D). Therefore, the results suggest that CK2 would not affect ER stress-induced induction of ATF6 cleavage.

### Expression and Subcellular Distribution of CK2

**Figure 7:**
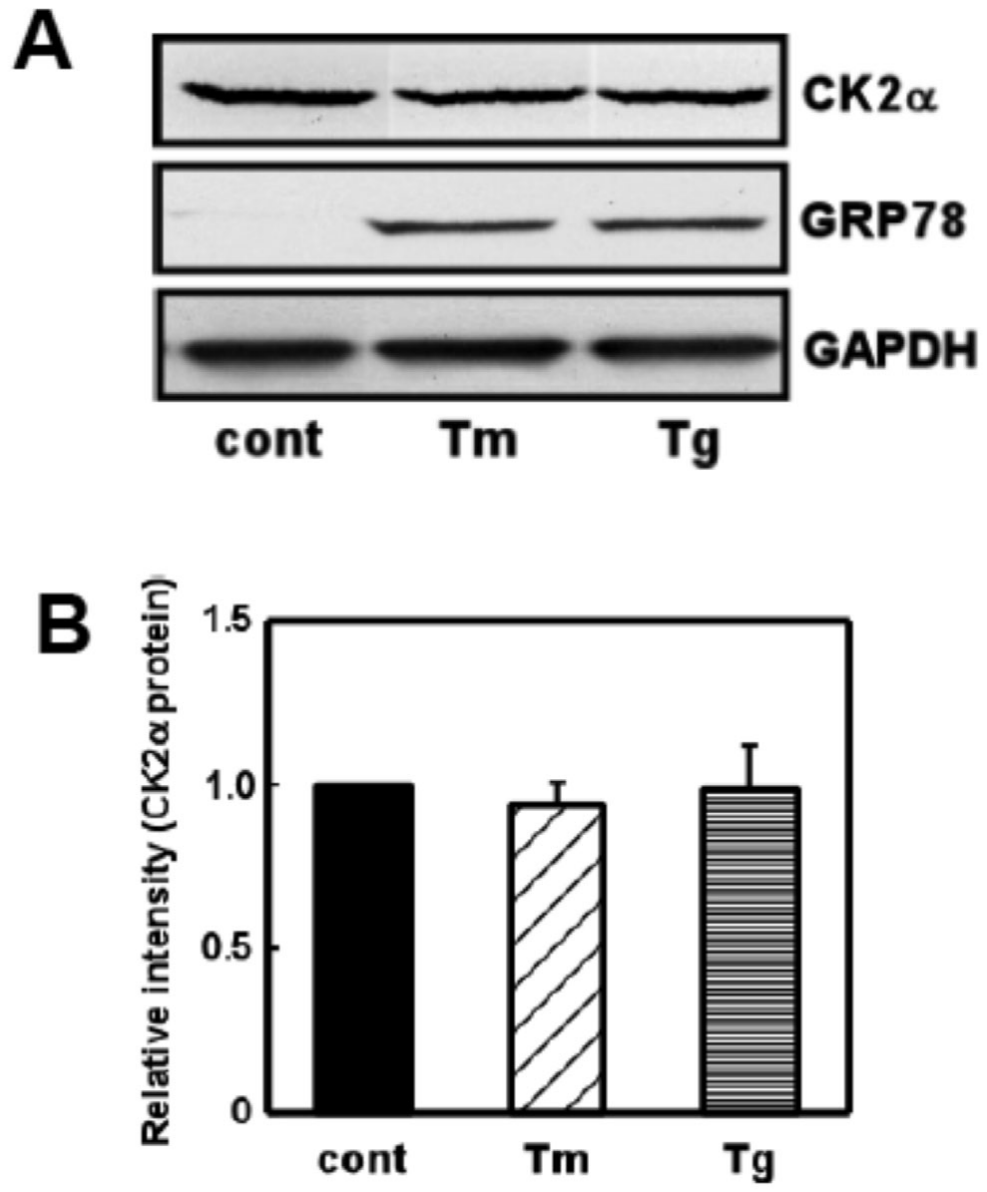
ER stress did not affect expression of CK2. (A) Primary cultured glial cells were treated with tunicamycin (Tm: 0.01 µg/mL) or thapsigargin (Tg: 0.01 µM) for 18 h and subjected to Western blotting. ER stress increased GRP78 levels whereas it did not affect levels of CK2. (B) Densitometric analysis of expression of CK2. n = 4/group. Results are expressed as the means ± S.E.

As we found that CK2 would contribute to ER stress-induced activation of UPR via the XBP-1-GRP78 pathway, we next investigated whether the expression of CK2 would be affected by ER stress. We used ER stress-inducing reagents (tunicamycin: Tm and thapsigargin: Tg) and analyzed the level of CK2 protein. Although both reagents markedly increased GRP78 levels, we did not observe the expression of CK2 (Fig. 7). The results suggest that ER stress would not affect the level of CK2. CK2 would therefore function to regulate XBP-1-GRP78 signaling by regulating its activity.

**Figure 8:**
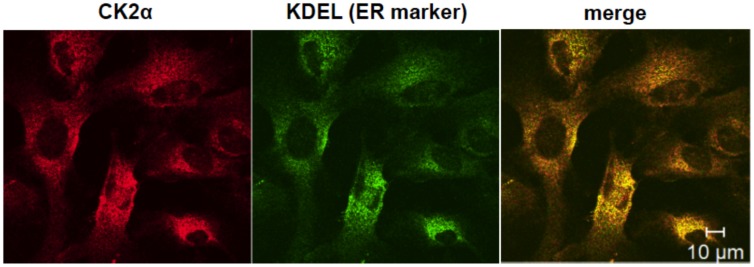
Existence of CK2 at the endoplasmic reticulum. Primary cultured glial cells were immunostained for anti-CK2 (left, red) and anti-KDEL (A marker of ER; middle, green) antibodies, respectively. The right panel is a merged image of the left and middle panels. Scale bar, 10 µm.

As CK2 can regulate an ER stress-activated UPR signal, the subcellular distribution of CK2 was analyzed by immunohistochemistry. We stained the ER with anti-KDEL antibody (ER marker) along with anti-CK2 antibody. Consistent with a previous report [19], the staining of CK2 merged with the ER marker (Fig. 8). The results suggest that CK2 exists in the ER in the glial cells.

## Discussion

Recent observations suggest the possible involvement of ER stress in several types of diseases, including neurodegenerative disorders, diabetes, obesity and cancer. However, the molecular regulatory mechanisms of ER stress are not fully understood. In the present study, we found CK2 to play a key role in regulating stress, which perturbs ER function.

Considering the linkage of XBP-1 on GRP78 induction, XBP1(s) was shown to induce GRP78 induction, as transfecting XBP-1 (s) plasmid increased GRP78 expression in HeLa cells [6]. However, tunicamycin-induced GRP78 expression was only modestly reduced in XBP-1-deficient MEF [20]. Therefore, contribution of XBP-1 on GRP78 induction under ER stressed-condition would be small. On the other hand, GRP78 expression was shown to be entirely ATF6α dependent [21]. In the present study, although the linkage of XBP-1 on GRP78 is small under ER stress [20], we found significant inhibition of both of the UPR component, i.e.; XBP-1 and GRP78, by inhibiting CK2. Moreover, although ATF6 processing was not affected by CK2 inhibitor, GRP78 was significantly inhibited in SH-SY5Y cells. So how would a modest effect on XBP1 splicing turn into a similar degree difference in GRP78 expression when ATF6 processing is intact? The reasons for these observations are at present unknown, but as CK2 has multiple substrates [22], it is possible that CK2 would affect other unknown UPR regulatory factor, which would affect GRP78 induction. ERdj4 is known to be regulated through XBP-1 under ER stressed condition [20]. In the preliminary study, as shown in the Figure S1, we found that ERdj4 was induced by ER stress in the primary cultured glial cells (Figure S1), suggesting that XBP-1 splicing may be functional in the glial cells. It would thus be interesting to investigate whether CK2 would affect ERdj4 similarly with XBP1 splicing and GRP78.

The involvement of CK2 in the control of UPR signaling is surprising and points to a novel mechanism for the regulation of ER stress under physiological conditions. For instance, CK2 would function to regulate immune reactions by connecting the cellular response to ER stress to inflammatory signaling. CK2 and p38 MAP kinase were shown to directly interact in response to TNFα [13], which itself can also activate UPR [23]. On the other hand, p38 MAP kinase phosphorylated a spliced form of XBP-1 at the residues Thr48 and Ser61, which subsequently enhanced its nuclear migration [24]. XBP-1 was shown to induce interleukin (IL)-6 expression [25]. We found that CK2 can activate ER stress-induced activation of XBP-1 splicing. Therefore, we can speculate from these observations that CK2 and XBP-1 would associate each other to regulate immune functions.

The present results may also have implications for the development of Alzheimer’s disease (AD). Several reports indicate the involvement of CK2 in the pathology of AD. It was reported that spermine-dependent CK2 activity was reduced by 84% and the amount of CK2 was reduced by 63% in the AD brain [26]. Amyloid β-protein stimulates CK2 activity, which would contribute to abnormal protein phosphorylation in the AD brain [27]. On the other hand, ER stress has been suggested to be involved in the pathology of AD in glial as well as neuronal cells [14], [28], [29]. These observations combined with the present result suggest the possible link between CK2 and ER stress with respect to AD.

In the present study, we provided new evidence of the mechanisms of ER stress; i.e. a key role for CK2 in regulating the XBP-1-GRP78 branch of UPR. By regulating such responses, cells would cope with stress. These basic responses would have important implications for regulating physiological events such as immune reactions, cell survival and cell death. Accumulating evidence has been suggested that neuron-glia communication to play an important role in the maintenance of normal CNS function. Therefore, it would be an important future subject to understand the role of CK2 on glial as well as neuronal cell function at the ER stressed condition. Furthermore, CK2 would also play a key role in the development of ER stress-related diseases such as neurodegenerative disorders, diabetes, obesity and cancer. Therefore, pharmacological manipulation of CK2 activity would be useful for treatments. Indeed, the use of CK2 inhibitors has been suggested to be useful for cancer therapy [30], [31]. Interestingly, an increased level of GRP78 expression was observed in tumors [32]. Therefore, targeting CK2 would inhibit the UPR activated in cancer cells, and be a new approach to treatment. Further understanding of the basic mechanism could be critical for unraveling the molecular mechanism/pharmacological treatment of ER stress-related disorders.

## Materials and Methods

### Materials

Tunicamycin (Tm) and thapsigargin (Tg) were obtained from Wako Pure Chemical Ltd. (Japan). 4,5,6,7-tetrabromobenzotriazole (TBB) [18] was obtained from Calbiochem.

### Preparation of Primary Cultured Glial Cells

Glial cells were prepared from the whole brains of neonatal C57BL/6 mice as described previously [33]. The cells were allowed to grow to confluence (10 days) in DMEM medium with 10% FCS, 100 units/ml penicillin G, and 100 µg/ml streptomycin. All cultured cells were kept at 37°C in 5% CO_2_/95% air. Subsequently, mixed glial cells were shaken at 120 rpm for 18 h, cultured again for 4 to 6 days in 35 mm dishes, and then used in the following experiments. At this point, astrocyte cultures were routinely >95% positive for glial fibrillary acidic protein, and ∼ 3% of the cells were microglia, based on positive ED1 (anti-macrophage/microglia monoclonal antibody) staining.

#### Ethics Statement

Animal experiments were carried out in accordance with the NIH Guide for Care and Use of Laboratory Animals and approved by the animal care and use committee at Hiroshima University (Permit number: A10–41).

### RNAi Experiment

Transient transfections of siRNAs were performed in 60–70% confluent primary cultured glial cells. Lipofectamine™ 2000 (Life technologies) was used to transfect siRNA according to the manufacturer’s directions. Opti-MEM1 medium was used for the transfection and the final concentrations of siRNA were 75 nM. CK2 has two isoforms: CK2α and CK2α’. Because of strong functional overlap between the two isoforms, we simultaneously knocked down CK2α and CK2α’ using the following siRNA sequences: CK2α; 5′-AAC AUU GAA UUA GAU CCA CGU dTdT-3′, CK2α’; 5′-AAG AUU CUG GAG AAC CUU CGU dTdT-3′. We used siPerfect Negative Control (SIGMA; SP-NEG) for the control siRNA transfection. Transfection efficiency of siRNAs for knocking down CK2 was approximately 55%. Cells were harvested 72 h after the transfection.

### Gene Expression Analysis

Total RNA was isolated using TRI Reagent (Sigma-Aldrich, St. Louis, MO, USA). Reverse transcriptase-polymerase chain reaction (RT-PCR) was performed as described previously [34]. cDNA was synthesized from 2 µg of total RNA by reverse transcription using 25 U of Superscript Reverse Transcriptase (Invitrogen) and 0.25 µg of Oligo(dt)12–18 primer (Invitrogen) in a 20-µl reaction mixture containing First-Strand Buffer (Invitrogen), 1 mM dNTP mix, 10 mM DTT, and 20 U of RNaseOUT Recombinant Ribonuclease Inhibitor (Invitrogen). Total RNA and the Oligo (dt) 12–18 primer were pre incubated at 70°C for 10 min prior to the reverse transcription. After incubation for 1.5 h at 46°C, the reaction was terminated by incubating samples for 15 min at 70°C. For PCR amplification, 1.2 µl of cDNA was added to 10.8 µl of a reaction mix containing 0.2 µM of each primer, 0.2 mM of dNTP mix, 0.6 U of Taq polymerase (Roche Diagnostics), and reaction buffer. PCR was performed in a DNA Thermal Cycler (MJ Research, PTC-220). The following primer sequences were used: GRP78; upstream, 5′–ctg ggt aca ttt gat ctg acg g-3′, and downstream, 5′-gca tcc tgg tgg ctt cca gcc att c-3′, CHOP; upstream, 5′–ccc tgc ctt tca cct tgg-3′, and downstream, 5′-ccg ctc gtt ctc ctg ctc-3′, XBP-1; upstream, 5′–cct tgt ggt tga gaa cca gg-3′, and downstream, 5′-cta gag gct tgg tgt ata c-3′, GAPDH; upstream, 5′-aaa ccc atc acc atc ttc cag-3′ and downstream, 5′-agg ggc cat cca cag tct tct-3′. The PCR products (10 µl) were resolved by electrophoresis in an 8% polyacrylamide gel in TBE buffer. The gel was stained with ethidium bromide and photographed under ultraviolet light. Each bands of the density was measured using Image J 1.37v (Wayne Rasband, NIH) software.

### Western Blotting

Cells were washed with ice cold PBS and lysed in a buffer containing 10 mM HEPES-NaOH (pH 7.5), 150 mM NaCl, 1 mM EGTA, 1 mM Na_3_VO_4_, 10 mM NaF, 10 µg/ml aprotinin, 10 µg/ml leupeptin, 1 mM PMSF, and 1% NP-40 for 20 min. The lysates were centrifuged at 15,000 rpm for 20 min at 4°C, and the supernatants were collected. The samples were boiled with laemmli buffer for 3 min, fractionated by SDS-PAGE, and transferred at 4°C to nitrocellulose membranes. The membranes were incubated with anti-KDEL (StressGen; diluted to 1∶1,000), anti-CHOP (Santa Cruz; diluted to 1∶500), anti-phospho (Ser51)-eIF2α (Cell Signaling; diluted to 1∶1,000), anti- eIF2α (Santa Cruz; diluted to 1∶1,000), anti-ATF6 (Santa Cruz; diluted to 1∶1,000), anti-CK2 (Santa Cruz; diluted to 1∶1,000) and anti-GAPDH (Chemicon; diluted to 1∶2000) antibodies followed by anti-horseradish peroxidase-linked antibody. Peroxidase was detected by chemiluminescence using an ECL system.

### Immunohistochemistry

Cells were fixed with methanol for 10 minutes at −20°C. After being washed with PBS, the cells were incubated with 5% normal bovine serum at 37°C for 1 h, and allowed to react with anti-CK2 (Santa Cruz; diluted to 1∶100) and anti-KDEL (StressGen; diluted to 1∶100) antibodies at 4°C overnight. The cells were then incubated with anti-goat immunoglobulin G antibody conjugated with Alexa 546 (1∶2000) and anti-mouse immunoglobulin G antibody conjugated with Alexa 488 (1∶2000) at 37°C for 1 h. The cells were visualized using confocal laser scanning microscopy. The confocal laser scanning microscopy was carried out at the Analysis Center of Life Science, Natural Science Center for Basic Research and Development, Hiroshima University.

### Statistics

Results are expressed as the means ± S.E. Statistical analysis was performed using Student’s *t*-test or paired *t*-test.

## Supporting Information

Figure S1 **Primary cultured glial cells were treated with tunicamycin (Tm: 1** **µg/mL) for 5 h, and subjected to RT-PCR analysis.** ERdj4 levels were significantly increased by ER stress. n = 6/group **p<0.01 compared with ER stress (Tm) alone. Results are expressed as the means ± S.E. ERdj4 specific primer pair: upper 5′-GCT GTG GAG AAG CTG CGT CGG-3′, lower 5′- ATC CTG GCG TGT GTG GAA GTG G-3′.
(TIF)
