# Supplementary figures and images for: Inhibition of Casein Kinase 2 Modulates XBP1-GRP78 Arm of Unfolded Protein Responses in Cultured Glial Cells
Source: PLoS One. 2012 Jun 29;7(6):e40144. doi: 10.1371/journal.pone.0040144 (PMC3387139; doi:10.1371/journal.pone.0040144)

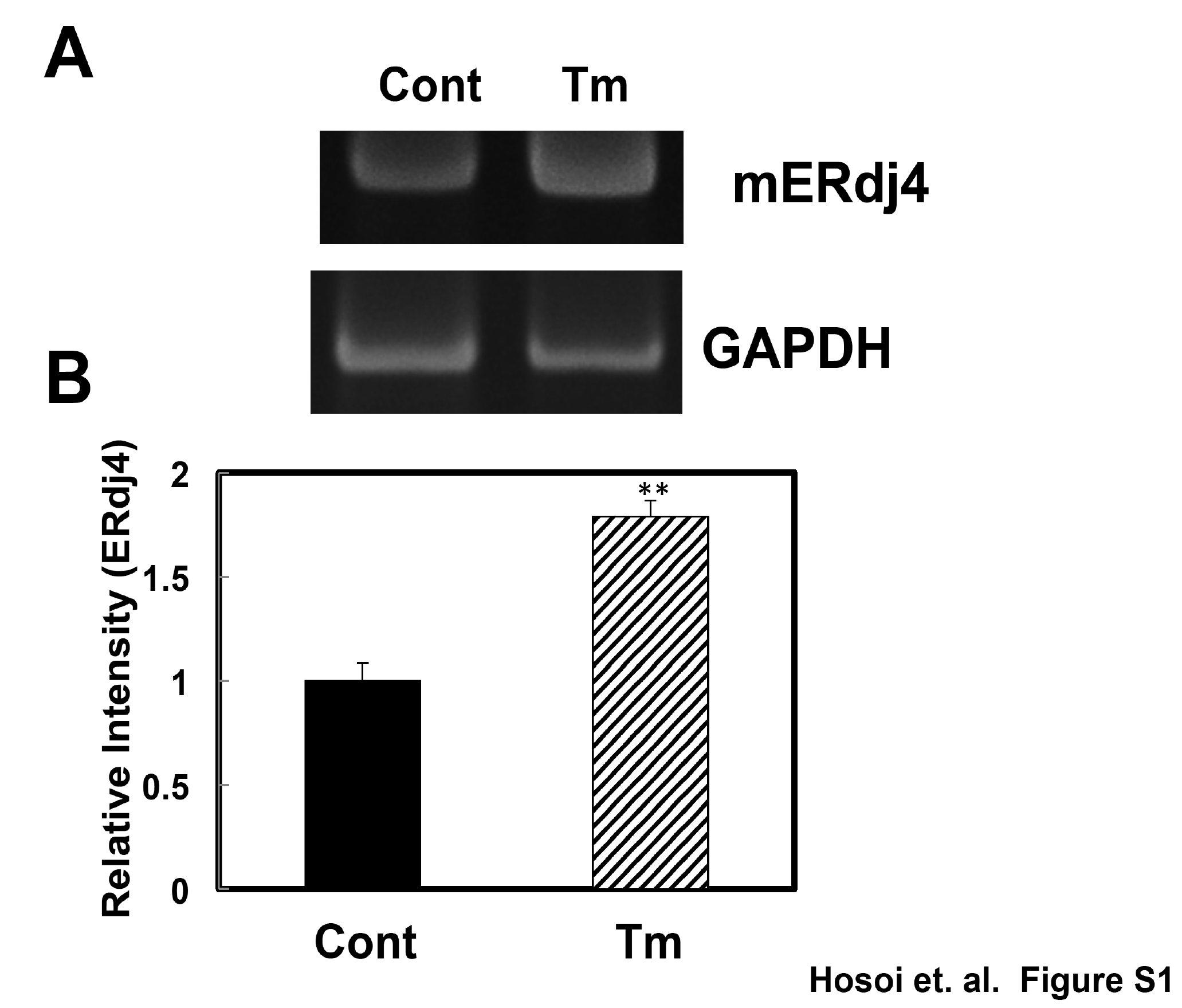

Supplement: Figure S1 — Primary cultured glial cells were treated with tunicamycin (Tm: 1 µg/mL) for 5 h, and subjected to RT-PCR analysis. ERdj4 levels were significantly increased by ER stress. n = 6/group **p<0.01 compared with ER stress (Tm) alone. Results are expressed as the means ± S.E. ERdj4 specific primer pair: upper 5′-GCT GTG GAG AAG CTG CGT CGG-3′, lower 5′- ATC CTG GCG TGT GTG GAA GTG G-3′. (TIF) [file pone.0040144.s001.tif]
